# THE IMPACT OF DIGITAL HEALTH LITERACY ON CANCER PREVENTION BEHAVIORS AMONG OLDER ADULTS

**DOI:** 10.1093/geroni/igae098.2316

**Published:** 2024-12-31

**Authors:** Jonathan Nutakor, Lulin Zhou, Ebenezer Larnyo, Stephen Addai-Dansoh

**Affiliations:** Jiangsu University, Zhenjiang City, Jiangsu, China (People’s Republic); Jiangsu University, Zhenjiang City, Jiangsu, China (People’s Republic); University of California, Santa Barbara, Santa Barbara, California, United States; Jiangsu University, Zhenjiang City, Jiangsu, China (People’s Republic)

## Abstract

In today’s age of digital advancement, it is crucial to understand how digital health literacy affects the cancer prevention behaviors of older adults. This study investigates how digital health literacy influences older adults’ ability to navigate online health information and its impact on adopting behaviors to prevent cancer. This study analyzes data from the Health Information National Trends Survey (HINTS) 5, focusing on individuals aged 60 and above. It explores different variables related to digital health literacy and engagement in cancer prevention behaviors, such as smoking cessation, physical activity, alcohol cessation, and cancer screening. Better digital health literacy is linked to an increased chance of quitting smoking (OR = 1.90, 95% CI: 1.02-3.52, p = 0.043). However, there is no such link to physical activity or cancer screening. Nevertheless, searching for health information electronically is associated with giving up alcohol, suggesting that digital health literacy can assist older adults in quitting drinking (OR = 1.47, 95% CI: 1.09-1.98, p = 0.012). The study highlights the importance of digital health literacy in promoting healthy habits among older adults. Specifically, it may help support smoking and alcohol cessation efforts. Though it may not apply to all behaviors, improving digital competencies is crucial to prevent cancer in older adults effectively. Therefore, tailored digital health literacy interventions are necessary to cater to the unique needs of this demographic and minimize their cancer risk.

